# Terrible Triad of the Elbow: Proposal and Reliability Validation of a Novel CT-Based Alphanumeric Classification System

**DOI:** 10.3390/jcm15208023

**Published:** 2026-10-16

**Authors:** Alessandra Colozza, Arianna Di Marcantonio, Antonino De Rosa, Carlotta Ventura, Sara Padovani, Biagio Zampogna, Edoardo Franceschetti, Pasquale Farsetti, Rocco Papalia

**Affiliations:** 1 Department of Orthopaedics and Traumatology, “Ospedale degli Infermi” of Faenza, 48018 Faenza, Italy; alessandra.colozza@auslromagna.it (A.C.); sara.padovani@auslromagna.it (S.P.); 2 Department of Orthopaedics and Traumatology, “ San Giovanni Addolorata Hospital”, 00184 Rome, Italy; dimarcantonioarianna@gmail.com; 3 Department of Orthopaedic and Trauma Surgery, Università Campus Bio-Medico di Roma, Via Alvaro del Portillo 21, 00128 Rome, Italy; carlotta.ventura@unicampus.it (C.V.); b.zampogna@policlinicocampus.it (B.Z.); e.franceschetti@policlinicocampus.it (E.F.); r.papalia@policlinicocampus.it (R.P.); 4 Research Unit of Orthopaedic and Trauma Surgery, Fondazione Policlinico Universitario Campus Bio-Medico, Via Alvaro del Portillo 200, 00128 Rome, Italy; 5 BIOMORF Department, Biomedical, Dental and Morphological and Functional Images, University of Messina, A.O.U. Policlinico “G. Martino”, 98125 Messina, Italy; 6 Department of Orthopaedics and Traumatology, “ Policlinico Tor Vergata” of Rome, 00133 Rome, Italy; farsetti@med.uniroma2.it

**Keywords:** terrible triad, posterolateral fracture-dislocation, elbow dislocation, radial head fracture, coronoid fracture, classification, reliability

## Abstract

**Background/Objectives**: The terrible triad (TT) of the elbow combines elbow dislocation with radial-head and coronoid fractures. Existing systems classify either the individual osseous components or the broader spectrum of elbow fracture-dislocations, requiring multiple labels to describe the combined TT morphology. We propose a novel combined alphanumeric CT-based classification that integrates radial-head and coronoid morphology into one TT-specific code. The rationale is not merely terminological: the two axes correspond to osseous features routinely considered during preoperative planning and may therefore provide a compact, clinically oriented description in addition to standardising communication and research stratification. **Methods**: Radial-head morphology was coded 1–3 using CT-derived displacement, comminution and radial-neck involvement, while coronoid morphology was coded A–C using tip-fragment height and basal extension. Among 167 consecutive TT patients treated between 2012 and 2023, 63 had a qualifying post-reduction CT and met all eligibility criteria. Three observers with different levels of expertise independently classified anonymised CT datasets twice, three weeks apart, with randomised case order at T2. Interobserver agreement among all three raters was assessed with Fleiss’ κ; pairwise Cohen’s κ and exact percentage agreement were secondary analyses, and intra-observer agreement was assessed with Cohen’s κ. **Results**: For the combined alphanumeric code, Fleiss’ κ increased from 0.708 (95% CI 0.584–0.815) at T1 to 0.817 (0.702–0.913) at T2, with exact three-rater agreement of 71.4% and 84.1%, respectively. Radial-head Fleiss’ κ was 0.761 (0.615–0.888) at T1 and 0.821 (0.682–0.940) at T2; coronoid κ was 0.746 (0.602–0.864) and 0.807 (0.676–0.912). Intra-observer κ for the combined code ranged from 0.753 to 0.886. The final descriptive distribution was 1B 2/63 (3.2%), 2A 5/63 (7.9%), 2B 8/63 (12.7%), 3A 19/63 (30.2%), 3B 27/63 (42.9%) and 3C 2/63 (3.2%); no 1A, 1C or 2C cases were present. **Conclusions**: The novel combined CT-based alphanumeric system offers a compact, reproducible descriptor of paired osseous morphology. Its added value lies in integrating established fracture concepts into one TT-specific code with prespecified CT rules. In addition to standardising communication and research stratification, the paired descriptor may provide a clinically intuitive framework for treatment planning by simultaneously conveying radial-head complexity and coronoid morphology. It is not intended to replace assessment of ligamentous injury, dynamic stability or intraoperative findings, and treatment-guiding and prognostic validity require dedicated outcome-based studies. Level of evidence: IV (reliability study).

## 1. Introduction

The terrible triad (TT) of the elbow is a complex injury pattern consisting of elbow dislocation together with a fracture of the radial head and fracture of the coronoid process. Popularised by Hotchkiss in 1996 [1], the combination was historically associated with poor outcomes and high rates of complications, such as chronic instability, stiffness and post-traumatic arthritis [2]. The label “terrible” reflected the disappointing functional results that followed surgical management, which frequently required revision. Because the injury results from a posterolateral mechanism, several authors now favour the descriptive term posterolateral fracture-dislocation; for consistency with the existing literature and for searchability, the established term terrible triad is retained throughout this manuscript.

Despite advances in surgical technique and fixation devices, TT injuries remain challenging because of the interplay between the osseous and ligamentous stabilisers of the elbow. Successful treatment requires restoring sufficient stability to allow early motion, which is critical to prevent stiffness [3]. If inadequately addressed, malreduction or overlooked fragments can compromise joint congruence and lead to debilitating sequelae [4].

Several classification systems describe individual elements of the injury (Table 1). Radial-head fractures are commonly reported with Mason/Hotchkiss-based systems [5], whereas coronoid fractures are described with Regan–Morrey or the morphology-based O’Driscoll system [6,7]. These systems remain clinically useful, but a TT injury is a combined lesion and is usually communicated using at least two separate osseous labels. Reliability studies have also shown observer variation: 3D CT improves agreement for radial-head and coronoid classifications but does not eliminate disagreement [8,9]. The present proposal addresses a different practical problem. Its added value is not simply the compression of two labels into one: it requires a standardised post-reduction CT assessment of both bony components using prespecified rules and produces a single TT-specific injury phenotype. This may facilitate handover, operative planning, consistent documentation, registry coding and comparative research. Importantly, the encoded features—radial-head displacement/comminution and radial-neck extension, together with coronoid tip size and basal involvement—are also variables routinely considered when planning reconstruction; the system was therefore intentionally designed to retain clinical and treatment relevance while remaining a morphology-based classification.

The Wrightington classification addresses the wider family of elbow fracture-dislocations using a column-based concept and links injury pattern to associated soft-tissue disruption and treatment strategy [10,11]. Its reliability has been assessed with CT, with interobserver κ values at approximately 0.71–0.73 in the original validation and moderate agreement in a recent external validation, again improving with combined 2D/3D CT [12,13]. The proposed alphanumeric system has a narrower but more granular aim: it is restricted to TT injuries and records the paired radial-head/coronoid osseous morphology in a single label. The systems are therefore complementary rather than competing. Wrightington frames the global instability pattern and inferred ligamentous injury, whereas the proposed code specifies the two bony problems that must be assessed and, when necessary, reconstructed. Two injuries that fall within the same broader TT category may therefore carry different alphanumeric codes and different osseous reconstructive challenges.

Treatment strategies for the TT have evolved substantially. Early protocols emphasised fixation of both fractures and repair of the lateral collateral ligament (LCL) [14], whereas more recent work suggests that not all coronoid fractures require fixation if stability can be restored after addressing the radial head and LCL [15]. Controversy also persists regarding radial head management: reconstruction is preferred when feasible, but replacement may be advantageous in comminuted fractures [16,17]. Reported complication rates remain significant, with post-traumatic arthritis affecting a substantial proportion of patients even after surgical repair [18]. Nevertheless, contemporary series and reviews report improved functional outcomes with protocol-based management, and adequate treatment of the radial head appears to be crucial for outcome [19,20,21,22].

The primary aim of this study was to assess the inter- and intra-observer reliability of a novel combined CT-based alphanumeric classification for TT injuries. The system was conceived both as a reproducible communication tool and as a clinically oriented morphology framework: the number encodes radial-head features relevant to reconstructive complexity, while the letter encodes coronoid morphology relevant to restoration of the anterior bony buttress. Reliability is the primary endpoint of the present manuscript. The study does not test whether the code mandates a specific operation or predicts outcome; nevertheless, these intended clinical applications form part of the rationale for developing and testing the classification. We hypothesised that the prespecified CT criteria would show substantial inter- and intra-observer agreement.

## 2. Materials and Methods

### 2.1. Study Design and Setting

This retrospective observational reliability study was conducted at a single high-volume referral centre for upper-limb trauma in Northern Italy (Ospedale degli Infermi, Faenza, Italy). Radiographic and CT images acquired between January 2012 and December 2023 as part of routine clinical care for elbow trauma were retrieved from the institutional imaging database and considered for analysis. The study was conducted in accordance with the ethical standards of the institutional research committee and with the principles of the Declaration of Helsinki and its subsequent amendments.

In accordance with applicable national legislation and institutional policy, formal Ethics Committee approval was not required because the study consisted exclusively of a retrospective evaluation of pre-existing, fully anonymised imaging data acquired during routine clinical practice, without any intervention, modification of patient management, or direct patient involvement.

All images were irreversibly anonymised by the hospital Information Technology Department prior to analysis. The investigators had no access to identifiable patient information at any stage of the study, and no identifiable data were collected, stored, or reported. No contact with patients occurred, no additional examinations were performed, and patient management was not influenced or modified in any way. Given the retrospective design and the exclusive use of fully anonymised data, the requirement for individual informed consent was waived, in accordance with Regulation (EU) 2016/679 (General Data Protection Regulation, GDPR), Recital 26, which excludes truly anonymised data from the scope of the Regulation.

### 2.2. Patient Selection

Patients were identified from the institutional trauma registry. Inclusion criteria were: (1) a confirmed diagnosis of TT, defined as a documented elbow dislocation associated with a fracture of both the radial head and the coronoid process (cases without a proven dislocation were not classified as TT and were excluded); (2) age ≥18 years; (3) availability of a post-reduction preoperative CT scan acquired at the authors’ centre after closed reduction in the dislocation; and (4) surgical management at the same institution.

Exclusion criteria were: (1) age < 18 years; (2) isolated elbow injuries not fulfilling all three TT components, including injuries without a proven dislocation; (3) prior elbow surgery that could alter bony anatomy or imaging interpretation; and (4) incomplete radiological data or absence of a qualifying preoperative CT.

Of the 167 patients initially identified, 104 were excluded, and 63 were analysed. The reasons for exclusion were *n* = 74 absence of a qualifying post-reduction CT scan performed at our institution; *n* = 18 injury not meeting all three TT criteria (absence of a documented elbow dislocation); *n* = 4 previous elbow surgery; *n* = 3 age < 18 years; and *n* = 5 incomplete radiological or clinical records. Baseline morphology was not available in a sufficiently complete form for the excluded group, so systematic differences between included and excluded patients cannot be excluded. In particular, the high proportion excluded because a qualifying post-reduction CT was unavailable may reflect differences in referral pathway, injury complexity or imaging practice and therefore represents a potential source of selection bias.

### 2.3. Imaging Protocol and CT Reconstruction

**Figure 1:**
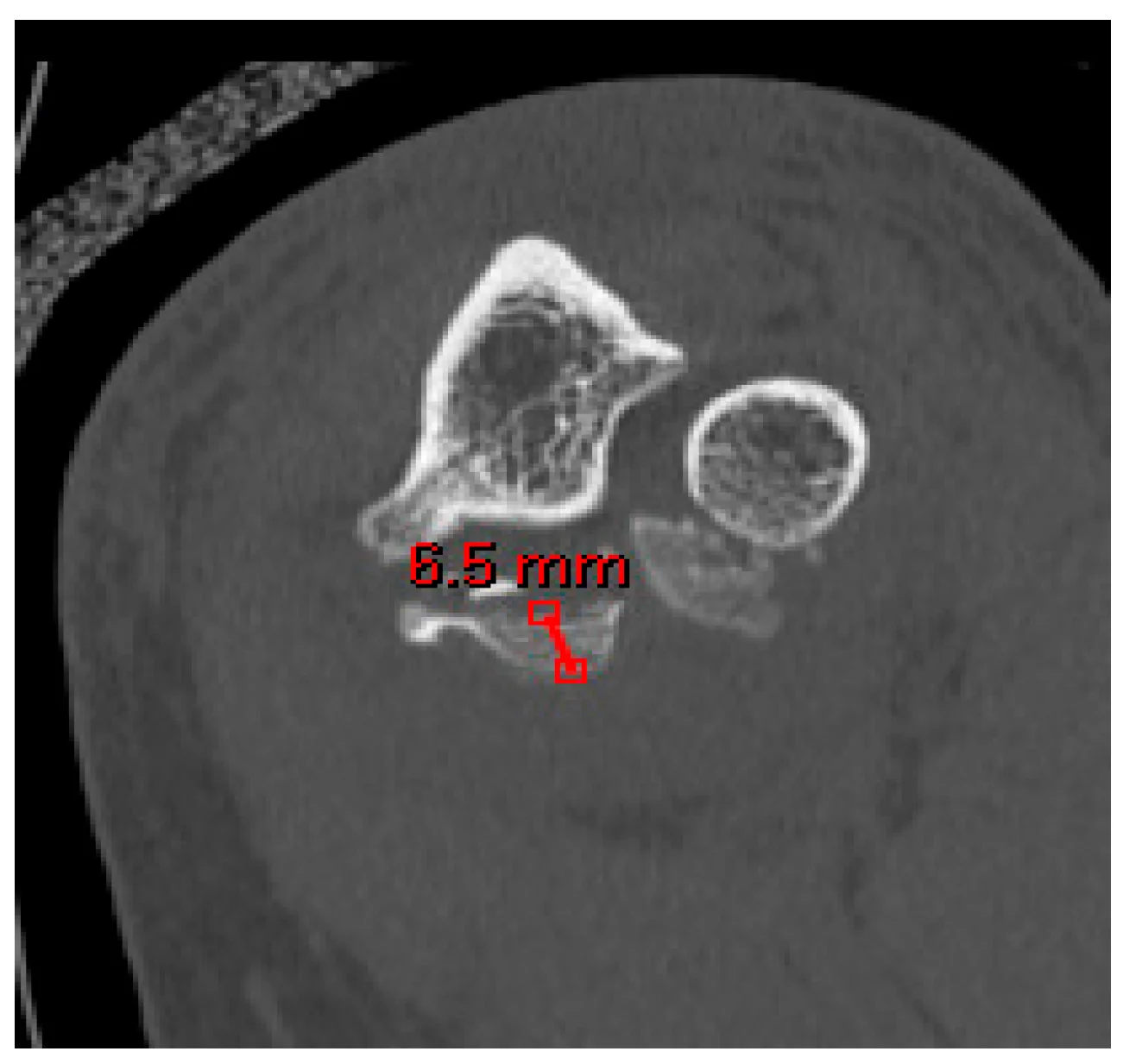
Coronoid assessment on CT. Coronal imaging is used to distinguish tip from basal morphology; fragment height is confirmed on a standardised sagittal reformat aligned with the ulnar shaft.

Following closed reduction in the dislocation, all included patients underwent multidetector CT of the elbow. A standardised reconstruction protocol was used so that classification did not depend on observer-generated reformats: slice thickness was 0.625 mm, with axial, sagittal and coronal multiplanar reformations and 3D volume-rendered reconstructions generated centrally. Radial-head morphology was assessed primarily on sagittal and axial 2D reformats with 3D correlation. Coronoid morphology was assessed in all three planes; the coronal plane was used to confirm tip versus basal extension, while the sagittal plane aligned with the ulnar shaft was used for fragment-height measurement (Figure 1). Observers were not permitted to create additional reformats during the reliability exercise.

### 2.4. Classification System

**Figure 2:**
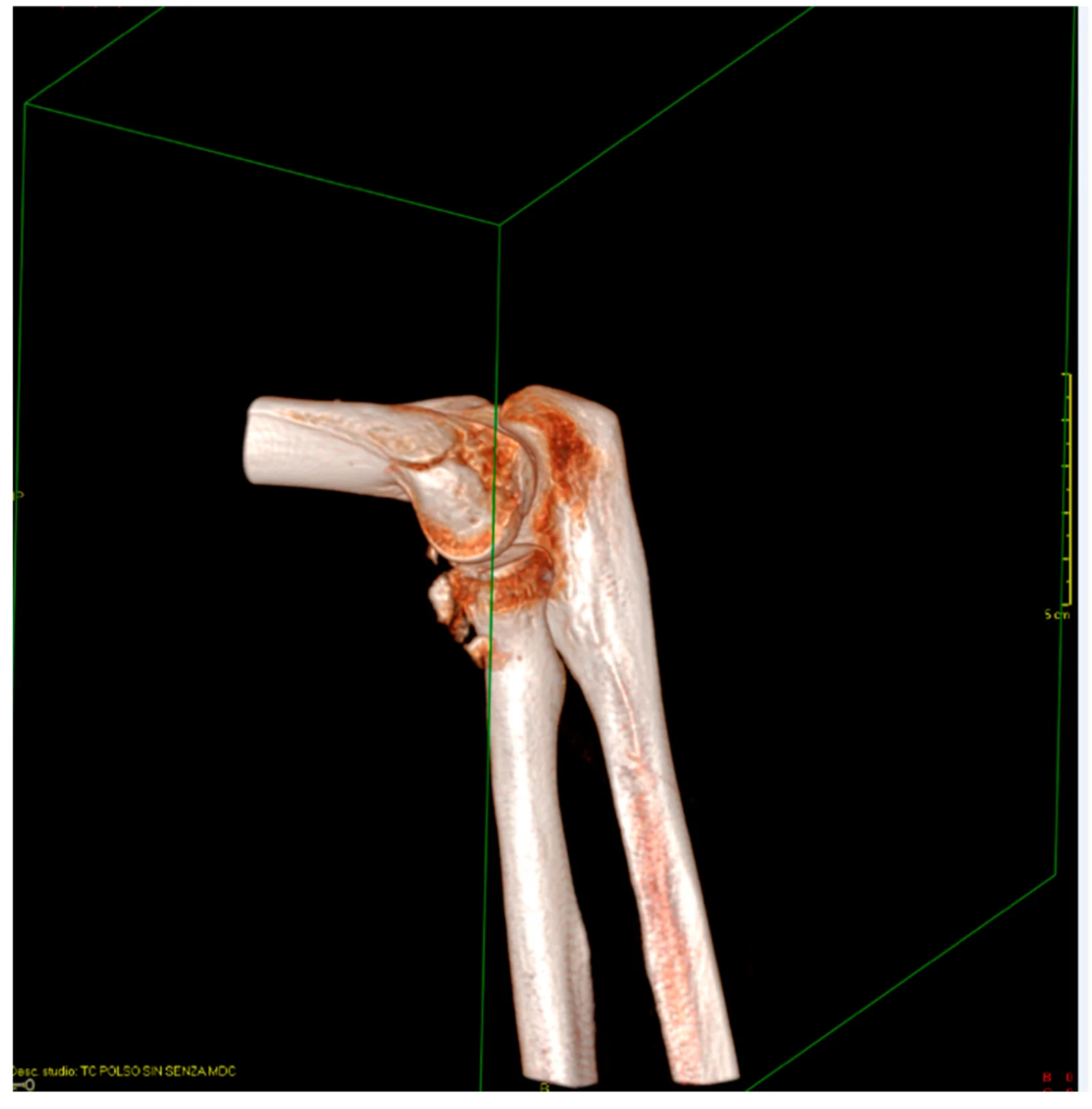
3D CT sagittal scanning was used for the measurement and analysis of the radial head fracture.

The classification combines a CT-based radial-head descriptor (1–3) with a CT-based coronoid descriptor (A–C). To ensure that the radial-head component is image-based, mechanical block and surgeon-dependent reconstructability were removed from the operational definition. Type 1 was defined as a non-comminuted radial-head fracture with <2 mm maximum articular displacement and no radial-neck extension. Type 2 was defined as a displaced but non-comminuted fracture with ≥2 mm maximum articular displacement and no radial-neck extension. Type 3 was defined by CT evidence of a multifragmentary radial-head fracture and/or extension into the radial neck (Figure 2). The 2 mm boundary is retained as a conventional Mason/Hotchkiss-derived morphologic threshold, but it is not interpreted as a validated treatment threshold because clinical outcome studies have questioned the use of displacement alone to determine operative treatment [23].

Coronoid fractures were graded as type A, type B or type C using prespecified CT landmarks. Type A was a tip fracture with maximal osseous fragment height ≤ 2.0 mm; type B was a tip fracture >2.0 mm; and type C was a basal fracture extending into the coronoid base/body. Fragment height was measured with workstation callipers on the standardised sagittal reformat as the maximal perpendicular distance from the osseous coronoid tip to the fracture line, with coronal/axial and 3D images used to confirm morphology. Exactly 2.0 mm was assigned to type A; values > 2.0 mm to type B. If basal extension was present, the fracture was classified as C regardless of tip height. The 2 mm A/B boundary was selected as a pragmatic, readily measurable CT threshold rather than as a proven biological cut-off. Its anatomical plausibility is supported by studies locating the proximal capsular attachment approximately 1.9–2.4 mm distal to the osseous coronoid tip [24,25], and a recent expert-consensus study also distinguished anterolateral tip fragments ≤2 mm from larger tip fragments [26]. The >50% coronoid-height component of the basal definition is consistent with the Regan–Morrey size concept and biomechanical evidence that loss of more than half the coronoid increases posterior translation [6,27].

### 2.5. Validation Procedure

Three independent observers with different levels of expertise—a senior elbow surgeon, a trauma surgeon and a senior orthopaedic resident—reviewed the anonymised CT datasets. The reliability exercise was image-based: the observers applied the same prespecified written category definitions and standardised CT planes and were not provided each other’s classifications during either reading. No discussion or consensus between raters was permitted before completion of T2. Each observer assessed all 63 cases twice, with a three-week interval; case order was randomised at T2 to reduce recall. The study records available for this revision do not document a separate formal calibration session or use of a reference atlas beyond the prespecified classification definitions and example images, and no such additional training is claimed. After the reliability analyses had been completed, a final descriptive classification was assigned for subtype-frequency reporting; this post hoc adjudication was not used in any κ calculation.

### 2.6. Statistical Analysis

Analyses were performed using the original statistical dataset in SPSS (version 29.0; IBM Corp., Armonk, NY, USA) and R (version 4.3; R Foundation for Statistical Computing, Vienna, Austria), with an additional re-analysis of the observer-level classification matrix for the present revision. The combined 1A–3C code was treated as a nominal paired descriptor rather than as a single ordinal severity scale; therefore, quadratic weighted κ and ICC were not used for the global classification. For interobserver agreement among all three raters, Fleiss’ κ was the primary statistic at T1 and T2, calculated separately for the combined alphanumeric code, the radial-head component (1–3), and the coronoid component (A–C). Pairwise Cohen’s κ values were reported secondarily for each observer pair. Intra-observer agreement between T1 and T2 was assessed with Cohen’s κ for each rater. Because κ can be influenced by imbalanced category prevalence, exact percentage agreement was reported alongside κ, and the distribution of all nine theoretical combined subtypes was explicitly reported. Ninety-five per cent confidence intervals were calculated for each reliability estimate; for the added Fleiss and combined-code analyses, subject-level nonparametric bootstrapping with 10,000 resamples was used. Agreement was interpreted using conventional κ benchmarks [28,29].

An a priori reliability sample-size calculation assumed a null κ of 0.40 and an expected κ of 0.80, with α = 0.05 and β = 0.20; a minimum of 54 cases was targeted. The final eligible cohort contained 63 cases. This calculation was a study-level feasibility target and does not guarantee adequate precision within rare individual subtypes. Continuous variables are reported as mean ± SD or median (IQR), and categorical variables as counts and percentages.

## 3. Results

### 3.1. Study Population

Over the 12-year period (2012–2023), 167 consecutive patients with an acute terrible triad injury were treated at the institution; after applying the inclusion and exclusion criteria, 63 were analysed (Table 2). The cohort comprised 31 women and 32 men with a mean age of 53 years (range 23–81).

### 3.2. Inter- and Intra-Observer Reliability

Intra-observer agreement was substantial to almost perfect. For the combined alphanumeric code, Cohen’s κ was 0.753 (95% CI 0.611–0.881) for the senior orthopaedic resident, 0.795 (0.663–0.910) for the trauma surgeon, and 0.886 (0.780–0.975) for the senior elbow surgeon. For the radial-head component, intra-observer κ values were 0.81, 0.84 and 0.96, respectively; for the coronoid component, they were 0.81, 0.74 and 0.87. Multi-rater interobserver agreement for the combined code was Fleiss’ κ = 0.708 (95% CI 0.584–0.815) at T1 and 0.817 (0.702–0.913) at T2. For the radial-head component, Fleiss’ κ was 0.761 (0.615–0.888) at T1 and 0.821 (0.682–0.940) at T2; for the coronoid component, 0.746 (0.602–0.864) and 0.807 (0.676–0.912), respectively. Exact agreement among all three raters increased from 71.4% to 84.1% for the combined code, from 85.7% to 90.5% for the radial head, and from 81.0% to 85.7% for the coronoid (Table 3). Pairwise Cohen’s κ results are shown separately (Table 4) because averaging pairwise coefficients is not used as the multi-rater estimate.

The inclusion of observers with different expertise levels was intended to test usability across experience strata rather than to establish a definitive expertise effect. All three observers achieved substantial or better intra-observer agreement for the combined code, with the highest value in the senior elbow surgeon (Table 5). Because only three observers were included, these findings should not be generalised to the broader orthopaedic community; validation with a larger and more diverse panel of raters is required.

### 3.3. Distribution of Injury Subtypes

**Figure 3:**
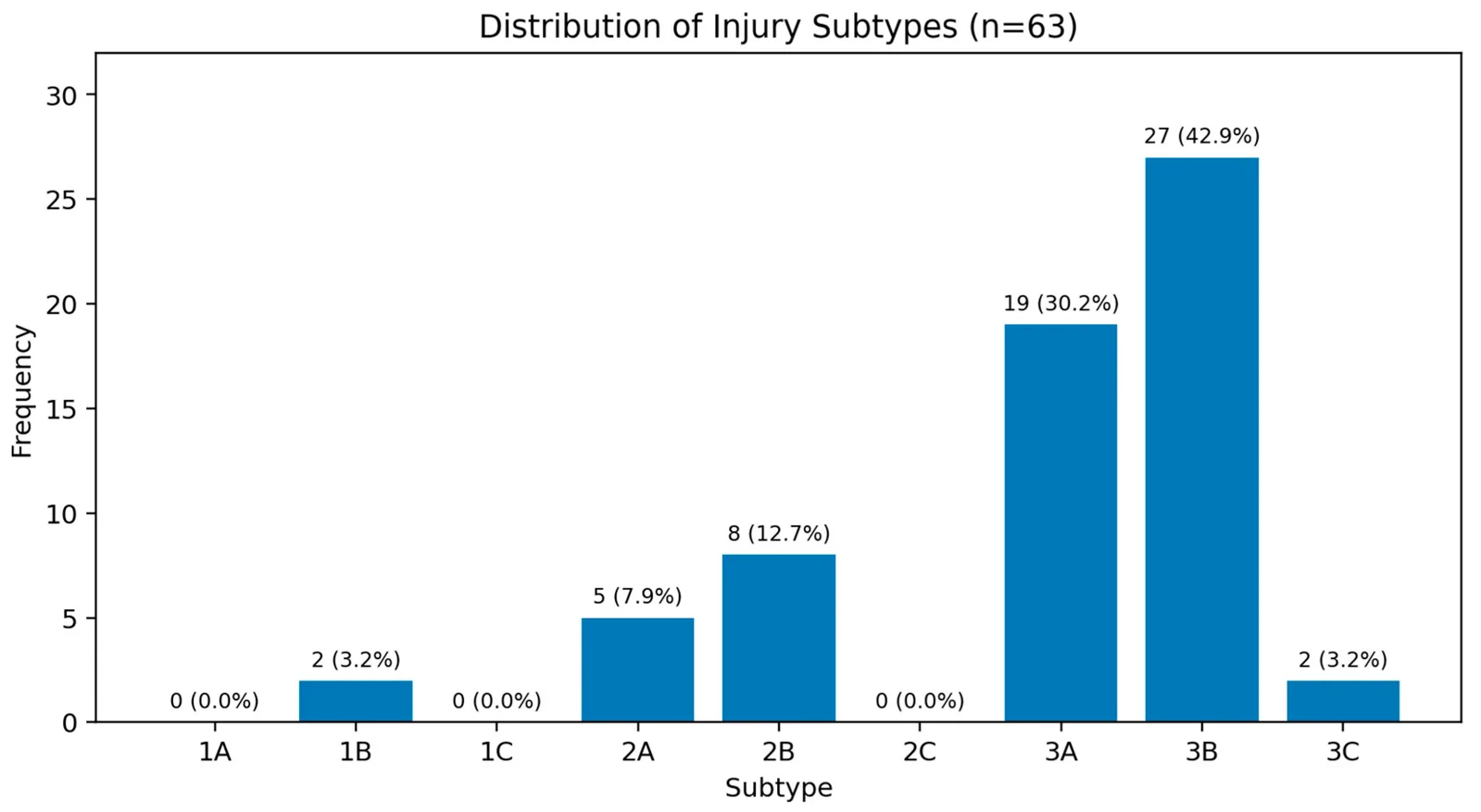
Distribution of the final terrible triad injury subtypes (*n* = 63). The most common patterns were 3B (27/63, 42.9%), 3A (19/63, 30.2%) and 2B (8/63, 12.7%). No 1A, 1C or 2C cases were present; 3C accounted for 2/63 (3.2%). Percentages do not total 100% because of rounding.

Using the final descriptive classification, the subtype distribution was: 1A 0/63 (0%), 1B 2/63 (3.2%), 1C 0/63 (0%), 2A 5/63 (7.9%), 2B 8/63 (12.7%), 2C 0/63 (0%), 3A 19/63 (30.2%), 3B 27/63 (42.9%), and 3C 2/63 (3.2%) (Table 6; Figure 3). Thus, three theoretical categories (1A, 1C and 2C) were absent, while the only observed type C pattern was 3C (2 cases). This pronounced prevalence imbalance is relevant when interpreting κ, because chance-corrected agreement can be influenced by rare categories; exact agreement is therefore reported alongside κ.

## 4. Discussion

This study evaluates the reliability of a novel combined CT-based alphanumeric descriptor for TT injuries. The data support substantial-to-almost-perfect agreement for the injury patterns represented in this cohort, particularly at the second reading. Because three theoretical subtypes were absent and type C injuries were rare, these findings should not be interpreted as a complete validation of the entire nine-subtype range. They do, however, support the feasibility and reproducibility of a single TT-specific code intended for communication, research stratification and clinically oriented description of the bony injury.

The principal contribution of the system is both pragmatic and clinical rather than merely terminological. A ‘3B’ label, for example, immediately communicates a multifragmentary/radial-neck radial-head pattern together with a >2 mm coronoid tip fragment, without requiring two independent classifications. This consolidation may reduce ambiguity in handover, referral and registry entry and may facilitate cohort aggregation by providing one reproducible variable. More importantly, radial-head and coronoid morphology are not independent pieces of information in TT management: they are considered together when restoring the radiocapitellar buttress and anterior ulnohumeral stability. The code therefore offers a concise preoperative morphology map of the two principal bony treatment problems. This does not mean that morphology alone determines treatment; rather, the system is intended to complement assessment of ligamentous injury, post-reduction stability, cartilage damage, patient factors and intraoperative findings.

These findings should be interpreted alongside the Wrightington system, which addresses a different level of the injury. Wrightington contextualises fracture-dislocations within a broader instability framework and incorporates inferred capsuloligamentous injury and treatment considerations [10,11]. Reliability studies report better agreement with CT than radiographs but still demonstrate observer variability, including a moderate agreement in external validation [12,13]. The proposed code does not directly encode ligamentous status, but it intentionally preserves granular bony information that is central to reconstruction. In clinical use, the two approaches can therefore be complementary: an instability framework defines the global lesion, while the alphanumeric code specifies the radial-head and coronoid morphology that may influence the osseous part of the surgical strategy.

The exclusion of the ligamentous injury is the most important conceptual limitation of the proposed system. Repair of the lateral collateral ligament and, in selected cases, the medial collateral ligament is central to restoring stability in the TT, and a classification intended to guide management should ultimately account for it. Future iterations should incorporate the ligamentous status, derived either retrospectively from operative notes or prospectively from preoperative imaging and intra-operative documentation, so that the descriptor reflects the full injury rather than its osseous component alone.

The 2 mm coronoid threshold warrants particular caution. It was chosen pragmatically to create a simple, reproducible CT boundary between very small tip fragments and larger tip fragments, not because 2 mm has been proven to represent a universal biomechanical or treatment threshold. Nevertheless, anatomical studies place the proximal capsular attachment approximately 1.9–2.4 mm distal to the osseous coronoid tip [24,25], and recent expert consensus separately considers anterolateral tip fragments ≤2 mm [26]. These data provide anatomical and contemporary clinical context for the chosen cut-off but do not validate it as a prognostic threshold. Similarly, the >50% component of type C is supported by established size-based classification and biomechanical work showing increased posterior displacement when more than half the coronoid is deficient [6,27]. Future studies should test measurement error at the boundary and compare absolute millimetric thresholds with size-normalised metrics.

The present manuscript is primarily a reliability study, and it cannot demonstrate that assigning a given code improves outcomes or that one operation is superior within a subtype. We do not, however, consider the treatment relevance of the classification to be purely theoretical. The two axes were intentionally built around morphology that already enters TT decision-making: radial-head displacement, comminution and neck extension influence the feasibility of fixation versus replacement, whereas coronoid tip size and basal extension influence the need and method of restoring the anterior buttress [14,15,16,17,19]. Accordingly, the combined code provides a concise preoperative description of the two bony problems that must be addressed. Definitive treatment remains dependent on ligamentous injury, dynamic stability after reduction and sequential reconstruction, cartilage damage, patient factors and intraoperative findings. We therefore retain a treatment-oriented table, but explicitly frame it as a literature-aligned, hypothesis-generating clinical interpretation rather than as an outcome-validated treatment algorithm (Table 7).

The reliability estimates are encouraging but should be interpreted in context. Multi-rater Fleiss’ κ for the combined code was 0.708 at T1 and 0.817 at T2, while exact three-rater agreement increased from 71.4% to 84.1%. Pairwise values were also reported to make the source of agreement transparent. The higher second-reading estimates may reflect familiarity with the classification, as well as reproducibility, and should not be interpreted as proof of a learning-independent performance advantage.

Limitations include the retrospective single-centre design; exclusion of 104/167 initially identified patients, mainly because an eligible post-reduction CT was unavailable; and the lack of sufficiently complete baseline morphology data for a formal included-versus-excluded comparison. Selection bias related to injury complexity, referral pathways or imaging practice therefore cannot be excluded. Only three observers participated, limiting generalisability across specialties and experience levels. The classification is intentionally osseous and does not encode ligamentous injury, so it must be used alongside clinical and intraoperative stability assessment. The 2 mm threshold is an operational definition rather than a validated biological cut-off. Three of the nine theoretical subtypes (1A, 1C and 2C) were absent from the final cohort and type C was represented only by two 3C cases; consequently, the study supports reliability only for the range of patterns actually observed, and prevalence imbalance may influence κ estimates. Finally, this reliability study did not test treatment efficacy or prognostic validity. The treatment-oriented interpretation in Table 7, therefore, represents the intended clinical use of the morphology and is grounded in the existing literature, not an outcome-validated recommendation. Prospective multicentre studies with larger observer panels, complete subtype representation and correlation with treatment choices and clinical outcomes are required.

## 5. Conclusions

The proposed novel combined CT-based alphanumeric classification provides a single, compact descriptor of radial-head and coronoid morphology in terrible triad injuries. In the patterns represented in this cohort, it demonstrated substantial-to-almost-perfect inter- and intra-observer agreement and may support more standardised communication, documentation and research stratification. Its added value extends beyond coding: by coupling two bony features that routinely influence reconstruction, it also offers a clinically intuitive, treatment-oriented preoperative morphology framework. The system is complementary to, rather than a substitute for, assessment of ligamentous injury, dynamic stability and intraoperative findings. Because three theoretical subtypes were absent, type C injuries were rare, only three observers were studied, and treatment/outcome validity was not directly tested, the present work establishes reliability of the observed spectrum rather than a validated therapeutic algorithm. Prospective multicentre and outcome-based validation is required to determine whether subtype assignment can predict treatment choice or clinical outcome.

## Figures and Tables

**Table 1:** Comparison of established elbow fracture classifications with the proposed combined TT descriptor.

Main Limitation for TT	Clinical Relevance	Published Reliability	Imaging Requirements	Anatomical Features	System
Radial head only; clinical mechanical block is not a CT feature	Widely used radial-head language	Mason-based interobserver agreement remains fair-to-moderate; 3D CT improves but does not eliminate disagreement	Radiographs; CT/3D CT improves morphology assessment	Radial-head displacement/comminution; clinical modifiers in Hotchkiss	Mason/Hotchkiss [5,8]
Coronoid only; height alone does not capture location	Simple size-based descriptor	κ ≈ 0.40 with 2D CT and 0.51 with 3D CT	Originally lateral radiograph; CT may supplement	Coronoid fragment height	Regan–Morrey [6,9]
Coronoid only; more complex and not a combined TT code	Morphology-based and injury-pattern oriented	κ ≈ 0.42 with 2D CT and 0.48 with 3D CT	CT, preferably multiplanar/3D	Coronoid morphology/location: tip, anteromedial facet, basal	O’Driscoll [7,9]
Less granular for paired radial-head/coronoid morphology within TT	Broad instability framework with treatment implications	Original: κ ≈ 0.71–0.73 with CT; external validation: moderate agreement, improved with 3D CT	Radiographs + 2D/3D CT	Whole elbow fracture-dislocation/column pattern with inferred soft-tissue injury	Wrightington [10,11,12,13]
Osseous only; does not replace ligament/dynamic stability assessment; rare subtypes underrepresented; treatment efficacy not outcome-validated	Single TT-specific code for communication, research stratification and treatment-oriented preoperative morphology	Current cohort: Fleiss κ 0.708 at T1 and 0.817 at T2 for observed patterns	Standardised post-reduction multiplanar CT + 3D reconstruction	Radial head 1–3 + coronoid A–C in one code	Proposed novel combined system

**Table 2:** Demographic and clinical characteristics of the study population (*n* = 63).

Variable	Value
Age (years)	53 ± 14.2 (range 23–81)
Sex	Female 31 (49.2%); Male 32 (50.8%)
BMI (kg/m^2^)	26.4 ± 3.8 (range 19–34)
Side	Right 35 (55.6%); Left 28 (44.4%)
Mechanism	Fall from standing height 27 (42.9%); high-energy trauma 36 (57.1%)

**Table 3:** Multi-rater interobserver reliability at T1 and T2. Fleiss’ κ is the primary nominal statistic; exact agreement denotes the proportion of cases for which all three raters assigned the same category.

T2 Exact Agreement	T2 95% CI	T2 Fleiss κ	T1 Exact Agreement	T1 95% CI	T1 Fleiss κ	Descriptor
84.1%	0.702–0.913	0.817	71.4%	0.584–0.815	0.708	Combined code (1A–3C)
90.5%	0.682–0.940	0.821	85.7%	0.615–0.888	0.761	Radial head (1–3)
85.7%	0.676–0.912	0.807	81.0%	0.602–0.864	0.746	Coronoid (A–C)

**Table 4:** Pairwise interobserver Cohen’s κ values (95% CI) for the combined code and the radial-head and coronoid components. Values are shown separately rather than averaged; the three-rater result is reported with Fleiss’ κ in Table 3.

Trauma vs. Senior Elbow Surgeon	Resident vs. Senior Elbow Surgeon	Resident vs. Trauma	Component/Reading
0.730 (0.582–0.862)	0.660 (0.499–0.799)	0.733 (0.594–0.859)	Combined code T1
0.793 (0.655–0.907)	0.820 (0.699–0.929)	0.839 (0.720–0.935)	Combined code T2
0.80 (0.65–0.95)	0.67 (0.48–0.86)	0.81 (0.64–0.97)	Radial head T1
0.78 (0.61–0.95)	0.84 (0.70–0.98)	0.84 (0.69–0.99)	Radial head T2
0.78 (0.62–0.93)	0.68 (0.50–0.86)	0.78 (0.63–0.93)	Coronoid T1
0.77 (0.62–0.93)	0.88 (0.76–0.99)	0.77 (0.61–0.93)	Coronoid T2

**Table 5:** Intra-observer Cohen’s κ for each rater. Combined-code confidence intervals were obtained by subject-level bootstrap; component-level values reproduce the prespecified statistical output.

Coronoid κ (95% CI)	Radial Head κ (95% CI)	Combined Code κ (95% CI)	Observer
0.81 (0.67–0.95)	0.81 (0.64–0.97)	0.753 (0.611–0.881)	Senior orthopaedic resident
0.74 (0.57–0.91)	0.84 (0.69–0.99)	0.795 (0.663–0.910)	Trauma surgeon
0.87 (0.75–0.99)	0.96 (0.88–1.00)	0.886 (0.780–0.975)	Senior elbow surgeon

**Table 6:** Distribution of the nine theoretical combined subtypes in the final descriptive classification (*n* = 63). Percentages do not total 100% because of rounding.

%	*n*	Subtype
0.0	0	1A
3.2	2	1B
0.0	0	1C
7.9	5	2A
12.7	8	2B
0.0	0	2C
30.2	19	3A
42.9	27	3B
3.2	2	3C

**Table 7:** Treatment-relevant interpretation of the classification. These considerations are literature-aligned and hypothesis-generating; they explain the clinical rationale of the descriptors but should not be interpreted as a validated treatment algorithm or as evidence that one treatment is superior within a subtype.

Descriptor	CT Morphology Conveyed	Potential Treatment Relevance (Literature-Aligned; Not Validated as an Algorithm)
Radial head 1	Non-comminuted; <2 mm articular displacement; no radial-neck extension	Lower osseous complexity. In TT, the radial head may require no or limited direct reconstruction if congruity and overall stability are restored; management remains stability-dependent [14].
Radial head 2	Displaced (≥2 mm) but non-comminuted; no radial-neck extension	Morphology generally amenable to reconstruction/ORIF when restoration of the radial head is required [14,16,17].
Radial head 3	Multifragmentary radial-head fracture and/or radial-neck extension	Greater reconstructive complexity; radial-head arthroplasty is commonly considered when stable anatomic fixation cannot be achieved [16,17,19].
Coronoid A	Tip fragment ≤ 2 mm without basal extension	Very small tip fragment; fixation may be unnecessary when stability is restored after radial-head and LCL management, but dynamic stability remains decisive [15].
Coronoid B	Tip fragment > 2 mm, remaining confined to the tip	Greater anterior bony involvement; fixation may be considered more readily when instability persists [14,15,26].
Coronoid C	Basal/body fracture; basal extension takes precedence, including >50% height involvement	Major structural involvement of the coronoid buttress; generally increases the need for fixation/reconstruction, with technique dictated by fragment morphology and overall instability [6,7,27].

## Data Availability

The anonymised data supporting the findings of this study are available from the corresponding author upon reasonable request. The data are not publicly available because they consist of clinical imaging records held by the participating institution.

## Abbreviations

The following abbreviations are used in this manuscript:

TT	Terrible triad
CT	Computed tomography
ICC	Intraclass correlation coefficient
CI	Confidence interval
LCL	Lateral collateral ligament
MCL	Medial collateral ligament
BMI	Body mass index
IQR	Interquartile range
SD	Standard deviation
GDPR	General Data Protection Regulation
